# *ATM* aberrations in chronic lymphocytic leukemia: del(11q) rather than *ATM* mutations is an adverse-prognostic biomarker

**DOI:** 10.1038/s41375-025-02615-5

**Published:** 2025-04-24

**Authors:** Birna Thorvaldsdottir, Larry Mansouri, Lesley-Ann Sutton, Ferran Nadeu, Manja Meggendorfer, Helen Parker, Christian Brieghel, Stamatia Laidou, Riccardo Moia, Davide Rossi, Jana Kotaskova, Julio Delgado, Ana E. Rodríguez-Vicente, Rocío Benito, Gian Matteo Rigolin, Silvia Bonfiglio, Lydia Scarfò, Mattias Mattsson, Zadie Davis, Panagiotis Baliakas, Inmaculada Rapado, Fatima Miras, Joaquín Martinez-Lopez, Javier de la Serna, Jesús María Hernández Rivas, María José Larráyoz, María José Calasanz, Karin E. Smedby, Blanca Espinet, Anna Puiggros, Lars Bullinger, Francesc Bosch, Bárbara Tazón-Vega, Fanny Baran-Marszak, David Oscier, Florence Nguyen-Khac, Thorsten Zenz, Maria Jose Terol, Antonio Cuneo, María Hernández-Sánchez, Sarka Pospisilova, Gianluca Gaidano, Carsten U. Niemann, Elias Campo, Jonathan C. Strefford, Paolo Ghia, Kostas Stamatopoulos, Richard Rosenquist

**Affiliations:** 1 Department of Molecular Medicine and Surgery, Karolinska Institutet, Stockholm, Sweden; 2 Institut d’Investigacions Biomèdiques August Pi I Sunyer (IDIBAPS), Barcelona, Spain; 3 Centro de Investigación Biomédica en Red de Cáncer (CIBERONC), Madrid, Spain; 4 MLL Munich Leukemia Laboratory, Munich, Germany; 5 Cancer Genomics, School for Cancer Sciences, Faculty of Medicine, University of Southampton, Southampton, UK; 6 Department of Hematology, Rigshospitalet, Copenhagen University Hospital, Copenhagen, Denmark; 7 Department of Hematology, Danish Cancer Institute, Copenhagen, Denmark; 8 Centre for Research and Technology Hellas, Institute of Applied Biosciences, Thessaloniki, Greece; 9 Division of Hematology, Department of Translational Medicine, University of Eastern Piedmont, Novara, Italy; 10 Clinic of Hematology, Oncology Institute of Southern Switzerland, Ente Ospedaliero Cantonale, Bellinzona, Switzerland; 11 Laboratory of Experimental Hematology, Institute of Oncology Research, Universita’ della Svizzera Italiana, Bellinzona, Switzerland; 12 Department of Internal Medicine, Hematology & Oncology, University Hospital Brno, Brno, Czech Republic; 13 Faculty of Medicine, Masaryk University, Brno, Czech Republic; 14 Central European Institute of Technology, Masaryk University, Brno, Czech Republic; 15 Cancer Research Center (IBMCC) CSIC-University of Salamanca, Salamanca, Spain; 16 Instituto de Investigación Biomédica (IBSAL), Salamanca, Spain; 17 Department of Hematology, University Hospital of Salamanca, Salamanca, Spain; 18 Hematology - Department of Medical Sciences, University of Ferrara, Ferrara, Italy; 19 IRCCS Ospedale San Raffaele, Milano, Italy; 20 Università Vita-Salute San Raffaele, Milano, Italy; 21 Department of Immunology, Genetics and Pathology, Uppsala University, Uppsala, Sweden; 22 Molecular Pathology Department, University Hospitals Dorset, Bournemouth, UK; 23 Hospital Universitario 12 Octubre, Madrid, Spain; 24 Spanish National Cancer Research (CNIO), Madrid, Spain; 25 Hematological Diseases Laboratory, CIMA LAB Diagnostics, University of Navarra, 31008 Pamplona, Spain, IdiSNA, Navarra Institute for Health Research, 31008 Pamplona, Spain; 26 Clinical Epidemiology Division, Department of Medicine Solna, Karolinska Institutet, Stockholm, Sweden; 27 Molecular Cytogenetics Laboratory, Pathology Department, Hospital del Mar and Translational Research on Hematological Neoplasms Group, Hospital del Mar Research Institute (IMIM), Barcelona, Spain; 28 Department of Hematology, Oncology and Cancer Immunology, Charité-Universitätsmedizin Berlin, corporate member of Freie Universität Berlin, Hum-boldt-Universität zu Berlin, Berlin, Germany; 29 Department of Hematology, Hospital Universitari Vall d’Hebron (HUVH), Experimental Hematology, Vall d’Hebron Institute of Oncology (VHIO), Department of Medicine, Universitat Autònoma de Barcelona, Barcelona, Spain; 30 Service d’hématologie biologique Hôpital Avicenne Assistance Publique des Hôpitaux de Paris Bobigny France, Bobigny, France; 31 Sorbonne Université, Service d’Hématologie Biologique, Hôpital Pitié-Salpêtrière, APHP, Paris, France; 32 Department of Oncology and Haematology, University Hospital and University of Zurich, Zurich, Switzerland; 33 Department of Hematology, INCLIVA Research Insitute, University of Valencia, Valencia, Spain; 34 Department of Biochemistry and Molecular Biology, Pharmacy School, Universidad Complutense de Madrid, Madrid, Spain; 35 Department of Clinical Medicine, University of Copenhagen, Copenhagen, Denmark; 36 Hospital Clínic of Barcelona, Barcelona, Spain; 37 Universitat de Barcelona, Barcelona, Spain; 38 Clinical Genetics and Genomics, Karolinska University Hospital, Solna, Sweden

**Keywords:** Chronic lymphocytic leukaemia, Cancer genomics

## Abstract

Despite the well-established adverse impact of del(11q) in chronic lymphocytic leukemia (CLL), the prognostic significance of somatic *ATM* mutations remains uncertain. We evaluated the effects of *ATM* aberrations (del(11q) and/or *ATM* mutations) on time-to-first-treatment (TTFT) in 3631 untreated patients with CLL, in the context of IGHV gene mutational status and mutations in nine CLL-related genes. *ATM* mutations were present in 246 cases (6.8%), frequently co-occurring with del(11q) (112/246 cases, 45.5%). *ATM*-mutated patients displayed a different spectrum of genetic abnormalities when comparing IGHV-mutated (M-CLL) and unmutated (U-CLL) cases: M-CLL was enriched for *SF3B1* and *NFKBIE* mutations, whereas U-CLL showed mutual exclusivity with trisomy 12 and *TP53* mutations. Isolated *ATM* mutations were rare, affecting 1.2% of Binet A patients and <1% of M-CLL cases. While univariable analysis revealed shorter TTFT for Binet A patients with any *ATM* aberration compared to *ATM*-wildtype, multivariable analysis identified only del(11q), trisomy 12, *SF3B1*, and *EGR2* mutations as independent prognosticators of shorter TTFT among Binet A patients and within M-CLL and U-CLL subgroups. These findings highlight del(11q), and not *ATM* mutations, as a key biomarker of increased risk of early progression and need for therapy, particularly in otherwise indolent M-CLL, providing insights into risk-stratification and therapeutic decision-making.

## Introduction

The protein kinase ataxia telangiectasia mutated (ATM) functions as a tumor suppressor, is activated in response to double-stranded DNA breaks, and plays a crucial role in DNA damage response [1]. In chronic lymphocytic leukemia (CLL), somatic mutations in *ATM* have previously been reported in 7–16% of cases [2–7], while the *ATM* gene, located in the q-arm of chromosome 11, is recurrently deleted in 10–24% of patients [3, 5, 6, 8–10]. Although del(11q) has long been recognized as a marker of poor prognosis in CLL and patients carrying this chromosomal abnormality frequently present with lymphadenopathy, face a higher risk of progression, and generally have an inferior prognosis [8, 9, 11–16], there are currently conflicting data on whether mutations in *ATM* with or without del(11q) are indicative of a poor clinical outcome.

In separate studies from the UK Leukemia Research Fund CLL 4 trial, patients with *ATM* mutations treated with chemotherapy exhibited shorter progression-free survival (PFS) and overall survival (OS) compared to *ATM-*wildtype patients, though the differences did not reach statistical significance [7, 17]. Of note, reduced survival was observed only in cases with biallelic *ATM* inactivation, defined as an *ATM* mutation combined with del(11q) [7, 17]. In contrast, Austen et al. demonstrated inferior OS and treatment-free survival in patients with *ATM* mutations [18], while Nadeu et al. identified *ATM* mutations, irrespective of del(11q), as a biomarker of shorter time-to-first-treatment (TTFT) but not OS in patients with CLL [4, 19]. More recently, Nguyen-Khac et al. observed that, although *ATM* mutations were associated with shorter PFS in univariable analysis, this association was lost in multivariable models when accounting for other recurrent CLL mutations [20]. Conversely, Hu et al. reported that *ATM* mutations remained a significant biomarker for shorter TTFT in both univariable and multivariable analyses in treatment-naive patients with CLL [21].

In a recent study, we investigated the prognostic impact of nine recurrently mutated genes, *ATM* not included, in a large well-annotated series of pre-treatment samples from 4580 patients with CLL [22]. We evaluated the relative clinical importance of mutations within each gene in the poor-prognostic immunoglobulin heavy variable (IGHV)-unmutated CLL (U-CLL) and the more favorable-prognostic IGHV-mutated CLL (M-CLL) subgroups separately, focusing on early-stage patients and TTFT. Notably, we reported that these recurrently mutated genes carry different prognostic impact depending on IGHV gene somatic hypermutation (SHM) status, indicating the need for a more compartmentalized strategy to enable tailored management and care for patients belonging to the M-CLL and U-CLL subgroups.

In the present investigation, which includes 3631 patients with CLL, we aimed to investigate the clinical impact of somatic mutations and/or deletions of *ATM*, with particular consideration of IGHV gene SHM status and other gene mutations associated with CLL prognosis, focusing primarily on early-stage patients. To our knowledge, this represents the largest study to date that investigates the prognostic implications of *ATM* dysfunction in this patient population, aiming to provide a comprehensive evaluation of its role in disease progression in relation to other genetic features.

## Materials and methods

### Patient cohort and clinicobiological characteristics

The present study included pre-treatment samples from 3631 patients with CLL collected from 22 European centers (Supplementary Table S1). This is a subset from the previously described cohort of 4580 patients for whom *ATM* mutational data was available [22]. The sample collection period spanned from 1996 to 2020 and the median time from diagnosis to sample collection was 3 months (IQR 0-34, data available for 3572 cases, 98.7%). The clinicobiological features of the analyzed cohort are outlined in Table 1 and are highly similar to those of the larger cohort, with no statistically significant differences [22]. In summary, the patients had a median age of 64.7 years at diagnosis, with a male-to-female ratio of approximately 2:1 (63% male). Moreover, 2604 patients (72%) were in Binet stage A, 1502 (44%) were categorized as U-CLL, and 2122 patients (58%) required treatment during follow-up of which the majority were treated with either chemotherapy (41%) or chemoimmunotherapy (38%) at first line, while only 1% received targeted therapy. All cases were diagnosed according to the iwCLL guidelines [23]. Informed consent was obtained according to the Helsinki declaration and the study was approved by the local Ethics Review Committees.

**Table 1:** Clinicobiological characteristics of the studied cohort (*n* = 3631).

		All cases	Binet A	Binet A M-CLL	Binet A U-CLL
**Gender**					
	**Male**	2281 (63%)	1530 (59%)	899 (58%)	540 (60%)
	**Female**	1350 (37%)	1074 (41%)	659 (42%)	358 (40%)
**Age at diagnosis (median years [IQR])**		64.7 [56.6-71.9]	65 [57.0-72.1]	65 [57.0-72.2]	64.7 [56.1-72.1]
**IGHV gene SHM status**					
	**M-CLL**	1900 (56%)	1558 (63%)		
	**U-CLL**	1502 (44%)	898 (37%)		
	**Unknown**	229	148		
Recurrent aberrations*					
	**del(13q)**	1458 (41%)	1128 (45%)	820 (54%)	233 (26%)
	**trisomy 12**	470 (13%)	314 (12%)	125 (8%)	175 (20%)
	**del(11q)**	412 (12%)	218 (9%)	33 (2%)	177 (20%)
	**del(17p)**	203 (6%)	132 (5%)	60 (4%)	66 (7%)
	**No recurrent abnormalities**	981 (28%)	738 (29%)	476 (31%)	233 (26%)
	**Unknown**	107	74	44	14
**Binet Stage**					
	**A**	2604 (72%)			
	**B**	716 (20%)			
	**C**	305 (8%)			
	**Unknown**	6			
Treatment status**					
	**Treated**	2122 (58%)	1186 (46%)	459 (29%)	676 (75%)
	**Untreated**	1509 (42%)	1418 (54%)	1099 (71%)	222 (25%)

*SHM* somatic hypermutation, *M-CLL* CLL with mutated IGHV genes, *U-CLL* CLL with unmutated IGHV genes, *TTFT* time to first treatment.

*According to the Döhner classification.

**During follow-up.

### Mutational analysis and variant classification

All cases had previously been assessed for coding sequence mutations in *BIRC3, EGR2, MYD88, NFKBIE, NOTCH1, POT1, SF3B1, TP53*, and *XPO1* [22]. Mutation screening for *ATM* was conducted using next-generation sequencing (NGS) in >99% of cases, primarily through the utilization of targeted gene panel sequencing, while only 22 cases were analyzed by Sanger sequencing or resequencing arrays (Supplementary Table S1). For cases investigated using NGS-based methods, sequence alignment, variant calling and annotation were performed at each participating center using a variant allele frequency (VAF) threshold of ≥5% to classify mutated cases. Additionally, polymorphic variants with a max population allelic frequency of ≥1% in the gnomAD database (v4.0) [24] were excluded, and the remaining variants were further filtered to only retain exonic non-synonymous variants and small insertions or deletions. All *ATM* variants are reported based on the reference transcript NM_000051.3/ENST00000278616.8 (GRCh37).

**Fig. 1:**
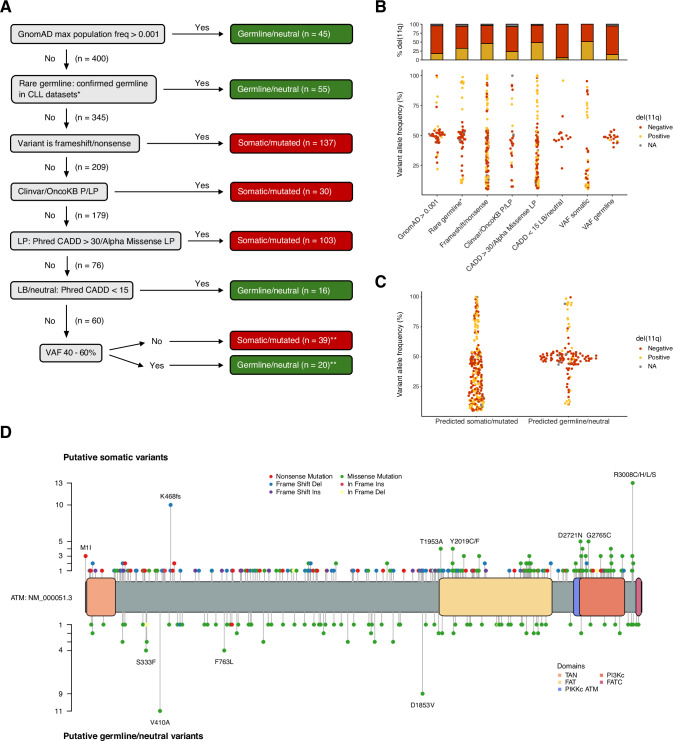
Classification of reported *ATM* variants. **A** Hierarchical flowchart used to assign *ATM* variants to putative ‘germline/neutral’ or ’somatic/mutated’ categories. **B** Distribution of variant allele frequencies (VAFs) for *ATM* variants in each flowchart category, bar chart shows the proportion del(11q) positive cases for each category. **C** Distribution of VAFs for all variants assigned as ‘germline/neutral’ and ‘somatic/mutated’. **D** Graphical representation of amino acid changes and frequencies for all *ATM* variants in the cohort. Putative somatic variants are displayed above and putative germline/predicted neutral variants are shown below. *Confirmed germline by sequencing in published datasets [30, 31] and in unpublished data. **Four *ATM* variants, each observed in multiple patients and variably classified as either germline/neutral or somatic/mutated depending on the final step of the classification system, were reviewed and reassigned to a single classification category (Supplementary Table S2).

*ATM* data were available from paired tumor/germline samples for 427 cases (11.8%), while tumor-only data were obtained for the remaining 3204 cases. Since the majority of sequencing results were derived from tumor-only analyses, capturing both somatic and germline variants, a hierarchical ranking system was implemented to classify all variants into categories of putative somatic or putative rare germline/predicted neutral (Fig. 1A). The classification of these variants was based on their max population allelic frequency ( > 0.001) in the gnomAD database (v4.0) [24], annotation in ClinVar [25], OnkoKB [26, 27], variant type (nonsense/frameshift or missense), pathogenicity predictions by AlphaMissense [28] and CADD (v1.7) [29], as well as data from CLL datasets with available germline information [2, 30, 31], and is detailed in Supplementary Table S2. For patients with multiple reported *ATM* variants, the variant with the highest hierarchical rank was assigned to each patient for subsequent analyses. If multiple variants fell within the same tier of the hierarchy, the variant with the highest VAF was prioritized.

The IGHV gene SHM status was available for 3402/3631 (93.7%) patients and was assessed using PCR amplification and sequence analysis of IGHV-IGHD-IGHJ gene rearrangements, utilizing a 98% identity threshold to germline sequences to define M-CLL (<98% identity) and U-CLL (≥98% identity) patients as previously described [32, 33]. Chromosomal aberrations were identified primarily through fluorescence in situ hybridization (FISH) and targeted probes for chromosomes 13q, 11q, 17p and 12 [11].

### Statistical analysis

Correlations between *ATM* mutational status and clinicobiological variables were assessed using the chi-squared test. Two-sided Fisher’s exact tests were performed to assess co-occurrence of genomic alterations and *p* values were adjusted using the Benjamini–Hochberg method for multiple testing. The primary endpoint for survival analysis was TTFT, calculated from the date of diagnosis until date of initial treatment or date of administrative censoring or death if untreated, with a median follow-up of 5.1 years (95% CI 4.7–5.5) and was available for 3598/3631 (99.1%) patients. Additionally, OS was assessed, calculated from the date of diagnosis to either the date of death or last follow-up. The cohort had complete OS data for all patients, with a median follow-up time of 13 years (95% CI, 12.2–13.6 years). Kaplan-Meier survival curves were calculated to evaluate the effects of *ATM* mutations and/or del(11q) on TTFT and OS. Pairwise comparisons were performed to determine differences between subgroups by employing the Cox–Mantel log-rank test and *p* values were adjusted using the Benjamini–Hochberg method to account for multiple comparisons. Multivariable analyses using Cox proportional hazards models were employed to assess the prognostic strength of individual biomarkers. All statistical analyses were performed in R (v4.4.1) [34] and R studio (version 2024.04.0 + 735) [35]. Plots were created using ggplot2 (version 3.5.1), survminer (version 0.4.9), ComplexHeatmap (version 2.20.0) and Maftools (version 2.20.0) [36].

## Results

### Hierarchical ranking of somatic and germline *ATM* variants

A total of 445 *ATM* variants were identified across 362 patients in the cohort studied following mutational analysis and initial variant filtering. Among these, 309 variants (69%) were classified as putative somatic using our proposed hierarchical ranking system for *ATM* variant classification. To validate this approach, we reclassified all confirmed somatic mutations in samples with available germline information. Of the 27 confirmed somatic *ATM* mutations, 25 were consistently classified as somatic, while 2 were reclassified as predicted neutral based on multiple pathogenicity scores suggesting a neutral impact (Supplementary Table S2). Furthermore, among variants reported as rare germline based on prior studies, only 3/55 were predicted to be pathogenic or likely pathogenic according to the ClinVar database, all three cases harbored del(11q) on the alternate allele.

As the next step, we plotted the VAFs for all variants assigned to each category in the flowchart (Fig. 1B). Variants classified as putative germline/predicted neutral typically had VAFs centered around 50%, whereas putative somatic mutations displayed a broader distribution with the majority falling below 50% (Fig. 1B and Supplementary Table S2). These distinct VAF patterns became even more pronounced when pooling all samples with putative germline/predicted neutral or putative somatic mutations (Fig. 1C). Notably, variants with VAFs >50% were most often observed in tumors also harboring del(11q) (Fig. 1B, C). We identified 17 *ATM* variants classified as putative somatic with VAFs >60% in patients without del(11q) (Supplementary Table S2). For three of these cases heterozygosity status was available, revealing copy number-neutral loss of heterozygosity (cnLOH) in all. For all subsequent analyses, all predicted germline/neutral *ATM* variants were re-categorized as *ATM* wildtype.

### Frequency of *ATM* mutations and association with clinicobiological features

Based on our classification, *ATM* mutations were identified in 246/3631 (6.8%) patients, with 57 (23.2%) of these individuals carrying more than one *ATM* mutation. The mutations were distributed across the entire coding sequence of the *ATM* gene, with the p.R3008C (*n* = 7, collectively p.R3008C/H/L/S *n* = 13) and the p.K468fs (*n* = 10) as the most frequently affected positions (Fig. 1D, Supplementary Table S3). Additionally, somatic missense mutations were primarily clustered toward the C-terminal region of the *ATM* protein, defined as the start of the FAT domain, while in contrast, somatic nonsense mutations were predominantly located in the N-terminal region (Fig. 1D and Supplementary Fig. S1). Putative germline/neutral variants were evenly distributed throughout the entire length of the *ATM* protein (Fig. 1D).

**Fig. 2:**
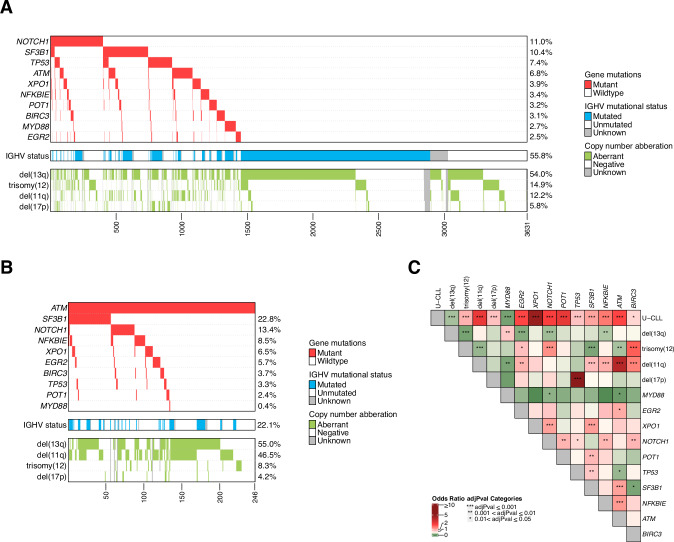
Overview of recurrent genetic aberrations in the entire cohort and *ATM*-mutated cases. **A** Overview of all 3631 CLL cases included in the study, sorted by frequency of mutations in 10 recurrently mutated genes. **B** Oncoplot showing detected mutations in recurrently mutated genes, IGHV somatic hypermutation status and chromosomal aberrations in 246 *ATM*-mutated cases. **C** Co-occurrence of recurrent gene mutations and chromosomal aberrations in the entire cohort. Odds ratios (OR) and BH-adjusted *p* values derived from two-sided Fisher exact tests. OR 0–1 indicates a trend towards mutual exclusivity, OR > 1 indicates a trend towards co-occurrence. U-CLL, CLL with unmutated IGHV genes, M-CLL, CLL with mutated IGHV genes.

**Fig. 3:**
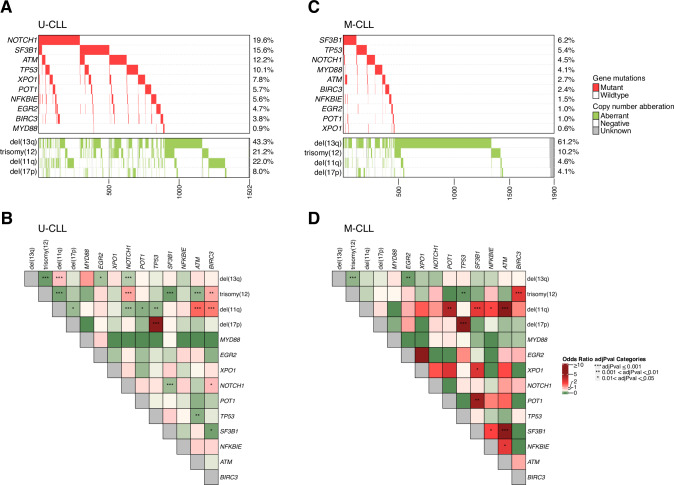
Overview of detected recurrent genetic abnormalities in U-CLL and M-CLL. **A** Oncoplot and (**B**) co-occurrence plot displaying recurrently mutated genes and chromosomal aberrations in 1502 U-CLL cases. **C** Oncoplot and (**D**) co-occurrence plot with recurrently mutated genes and chromosomal aberrations in 1900 M-CLL cases. Odds ratios (OR) and BH-adjusted *p* values derived from two-sided Fisher exact tests. OR 0–1 indicates a trend towards mutual exclusivity, OR > 1 indicates a trend towards co-occurrence. U-CLL CLL with unmutated IGHV genes, M-CLL CLL with mutated IGHV genes.

By combining our findings on *ATM* with sequencing data for nine other commonly mutated genes in CLL [22], *ATM* emerged as the fourth most frequently mutated gene, following *NOTCH1*, *SF3B1*, and *TP53* (Fig. 2A). Mutations in *ATM* frequently co-occurred with other gene mutations, observed in 134 of 246 cases (54%). Among these, mutations in *SF3B1* were the most common co-existing event, identified in 56 cases (23%, Fig. 2B). On the contrary, mutations in *TP53* and *MYD88* were rare occurrences in cases with *ATM* mutations, displaying mutual exclusivity (Fig. 2B, C). As expected, *ATM* mutations frequently co-occurred with del(11q) (112/246, 45.5%, Fig. 2B, C), while among cases with del(11q) (430/3631 cases, 11.8%), 112/430 (26.0%) carried *ATM* mutations. In addition, *ATM* mutations were significantly more frequent in U-CLL compared to M-CLL (*p* < 0.001), in patients with advanced stage at diagnosis (*p* < 0.001) and in those eventually in need of treatment (*p* < 0.001, Table 2). Notably, in U-CLL, *ATM* mutations were mutually exclusive with trisomy 12 and *TP53* mutations (Fig. 3A, B), while *SF3B1* and *NFKBIE* mutations were enriched in M-CLL (Fig. 3C, D).

**Table 2:** Relation of *ATM* mutations to other clinicobiological markers.

		*ATM*-mutated cases, *n* (%)	OR (95% CI)	*p* value
**Gender**				
	Female	85/1350 (6.3%)		
	Male	161/2281 (7.1%)	1.13 (0.86–1.50)	0.38
**IGHV status**				
	M-CLL	52/1900 (2.7%)		
	U-CLL	183/1502 (12.2%)	4.93 (3.62–6.82)	<0.001*
**Clilnical stage**				
	Binet A	147/2604 (5.6%)		
	Binet B/C	99/1021 (9.7%)	1.79 (1.37–2.34)	<0.001*
**Need for treatment**				
	untreated	50/1509 (3.3%)		
	treated	196/2122 (9.2%)	2.97 (2.18–4.12)	<0.001*
**del(11q)**				
	no del(11q)	129/3084 (4.2%)		
	del(11q)	112/430 (26.0%)	8.07 (6.10–10.66)	<0.001*
**del(17p)**				
	no del(17p)	231/3314 (7%)		
	del(17p)	10/203 (4.9%)	0.69 (0.34–1.26)	0.26
**trisomy 12**				
	no trisomy 12	220/2981 (7.4%)		
	trisomy 12	20/523 (3.8%)	0.50 (0.30–0.78)	0.003*
**del(13q)**				
	no del(13q)	108/1612 (6.7%)		
	del(13q)	132/1894 (7.0%)	1.04 (0.80–1.36)	0.75

*Statistically significant (*p* < 0.05), Chi-squared test.

### *ATM* aberrations and clinical outcome

We first evaluated the clinical impact of our *ATM* variant classification. Patients with putative germline/predicted neutral variants showed no significant differences in TTFT compared to *ATM-*wildtype cases while in contrast, cases with somatic *ATM* mutations displayed significantly shorter TTFT (Supplementary Fig. S2A–C). Results were also consistent when stratifying by del(11q) or limiting to Binet A cases (Supplementary Figs. S2D–F and S3), validating our re-categorization of germline/neutral variants with *ATM-*wildtype cases.

**Fig. 4:**
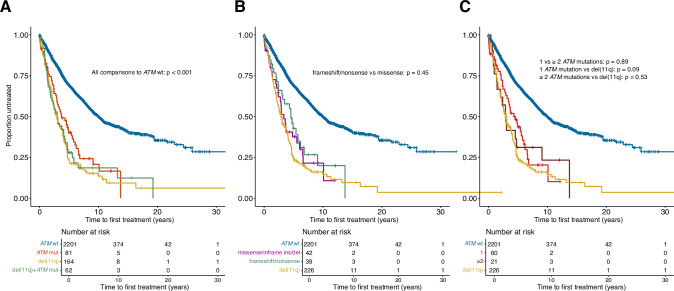
Clinical impact of *ATM* aberrations in Binet A CLL patients. Kaplan-Meier survival analysis using TTFT subdivided by (**A**) *ATM* aberration; somatic *ATM* mutation only, del(11q) only and combined *ATM* mutation and del(11q), (**B**) type of *ATM* mutation; frameshift/nonsense, missense/inframe indels with del(11q) displayed separately, and (**C**) comparing one versus multiple *ATM* mutations with del(11q) displayed separately.

We assessed the clinical impact of *ATM* aberrations on TTFT by investigating del(11q), *ATM* mutations as well as bi-allelic *ATM* abnormalities (del(11q) and *ATM* mutations) independently in Binet A patients (2604/3631, 72% of the cohort). Here, any type of *ATM* abnormality resulted in significantly shorter TTFT when compared to *ATM-*wildtype cases (Fig. 4A). Among those with *ATM* mutations, no differences were detected when comparing missense versus nonsense/frameshift mutations (Fig. 4B and Supplementary Fig. S4) or when comparing mutations located in the C- versus N-terminal (Supplementary Fig. S5). We also investigated whether carrying multiple *ATM* mutations, as opposed to a single mutation, affected clinical outcome and found no evidence for a shorter TTFT in the overall cohort or in Binet A cases specifically (Fig. 4C and Supplementary Fig. S6).

**Fig. 5:**
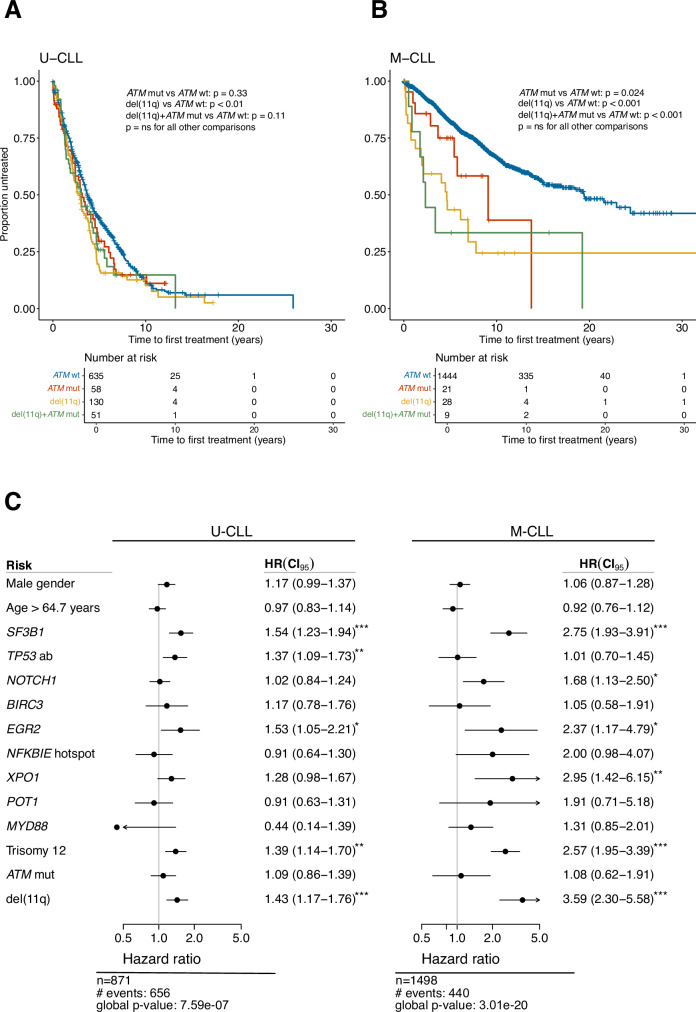
Clinical impact of *ATM* aberrations in Binet A CLL patients stratified by IGHV somatic hypermutation status. Kaplan-Meier survival analysis using TTFT in Binet stage A in (**A**) U-CLL and (**B**) M-CLL patients. Pairwise comparisons were performed using the Cox–Mantel log-rank test. **C** Multivariable analysis using TTFT in Binet stage A CLL patients with U-CLL and M-CLL. U-CLL, CLL with unmutated IGHV genes, M-CLL, CLL with mutated IGHV genes. CI95, 95% confidence interval; * indicates a *p* value < 0.05, ** *p* < 0.01, and *** *p* < 0.001.

As we previously have shown that the prognostic impact of recurrent gene mutations in CLL is highly dependent on IGHV gene SHM status [22], we investigated if *ATM* mutations affected clinical outcome when patients were stratified into the U-CLL and M-CLL subgroups, again focusing primarily on Binet stage A CLL. Among patients with unmutated IGHV genes, only those carrying del(11q) but not *ATM* mutations had a significantly inferior TTFT when compared to *ATM-*wildtype cases (Fig. 5A). The same analysis in M-CLL cases revealed instead that any *ATM* abnormality, either mono-allelic, with or without del(11q) or bi-allelic, resulted in significantly shorter TTFT (Fig. 5B).

Since mutations in *ATM* and *SF3B1* often co-occurred, we also assessed if these aberrations in combination further affected TTFT. We noted no significant difference when comparing those carrying aberrations in *ATM* with or without an additional *SF3B1* mutation (Supplementary Fig. S7). Furthermore, isolated *ATM* mutations (without other poor prognostic genetic markers) were rare, occurring in 1.2% of all Binet A cases and in 0.7% of Binet A M-CLL cases specifically. Of note, in Binet A patients, isolated *ATM* mutations did not have a significant impact on TTFT in either the U-CLL or M-CLL subgroups (Supplementary Fig. S8).

As the next step, we aimed to assess the specific contribution of *ATM* mutations on TTFT by employing a multivariable model. In addition to *ATM* mutations, the model included nine recurrently mutated genes [22], patient gender, age at diagnosis, del(11q) and trisomy 12 status, and IGHV gene SHM status. Patients carrying somatic mutations in *TP53*, or harboring del(17p), were combined as *TP53* aberrant, while only cases carrying the hotspot mutation p.Y254fs/p.254* were considered mutated for *NFKBIE* [22]. Among Binet A cases, IGHV gene SHM status was the strongest significant biomarker of shorter TTFT, exhibiting the highest hazard ratio (Supplementary Fig. S9). In addition, mutations in *SF3B1, EGR2, XPO1, TP53* aberrations, trisomy 12 and del(11q), but not *ATM* mutations, independently predicted shorter TTFT (Supplementary Fig. S9). In separate multivariable models for Binet A patients, stratified by IGHV status, mutations in *SF3B1*, *TP53* aberrations, del(11q), trisomy 12 as well as *EGR2* mutations were the only significant independent variables in U-CLL (Fig. 5C). For M-CLL patients, mutations in *SF3B1*, *NOTCH1*, *XPO1*, *EGR2*, trisomy 12 as well as del(11q) were significant biomarkers (Fig. 5C). *ATM* mutations had no independent significant effect on TTFT in either model.

Finally, we investigated the impact of different types of *ATM* mutations on OS across the entire cohort. Patients were stratified into four distinct groups based on their *ATM* status: *ATM*-wildtype, *ATM*-mutated, those carrying del(11q), and patients with biallelic alterations (del(11q) and *ATM* mutations). Any *ATM* aberration was associated with significantly reduced OS in the entire cohort and specifically among M-CLL patients. In contrast, within the U-CLL subgroup, only patients with del(11q) showed a significantly inferior outcome compared to those with wildtype *ATM* (Supplementary Fig. S10A–C). In multivariable analysis, del(11q), but not *ATM* mutations, emerged as a significant predictor of OS (Supplementary Fig. S10D–F).

## Discussion

In the current study, we aimed to evaluate the prognostic significance of *ATM* mutations in CLL and their impact within the context of IGHV gene SHM status and other CLL-associated gene mutations, using data from a large, well-characterized, multi-center European cohort. Given that a large proportion of cases in the studied cohort included tumor-only data, we developed a hierarchical ranking system which integrates published data from databases on both cancer-associated variants and variants present in normal populations, enabling the classification of mutations as putative somatic or germline/neutral. Using our classification, we could accurately assign variants in samples within our dataset with available germline information. Putative somatic variants exhibited markedly different VAF distributions compared to those classified as putative germline or predicted neutral, with the majority of somatic variants showing frequencies <50% while those with VAFs >50% were most often detected in patients with del(11q). Notably, we identified 17 variants classified as putative somatic in patients without del(11q), all exhibiting VAFs >60%. In three of these cases, the elevated VAFs were attributable to cnLOH, consistent with what has been previously reported in CLL [37]. Finally, in concordance with a previous report, somatic missense mutations were predominantly found at the C-terminal domains while frameshift and nonsense mutations mainly affected the N-terminal [38].

We used TTFT as the primary clinical endpoint to evaluate how our classification of *ATM* variants affected clinical outcome and found cases harboring putative somatic variants to have a significantly inferior outcome in univariate analyses compared to those carrying putative germline/neutral variants, with the latter group exhibiting results comparable to those of *ATM-*wildtype cases. While our flowchart seems to effectively distinguish patients with somatic mutations from those classified as germline/putatively neutral in our analysis of TTFT, we cannot entirely exclude the possibility that some of the variants classified as somatic might be rare pathogenic germline mutations or that variants classified as neutral may in fact impact prognosis through disruption of normal ATM function. Additionally, selected germline variants excluded from our analysis may still influence tumor development in CLL, as suggested by recent publications [30, 31]. Ultimately, as a result of our classification, 246/3631 (6.8%) patients of our cohort had *ATM* mutations, while 57/246 cases (23.2%) carried multiple *ATM* mutations. The frequency observed is slightly lower than reported in previous studies and might be attributed to differences in classification strategies and the potential inclusion of germline or predicted neutral variants in other studies.

We noted that mutations in *ATM* often co-occurred with other recurrent abnormalities in CLL, predominantly del(11q) (45.5%) and *SF3B1* mutations (23%). The former combination is well-known in CLL [7, 18, 39, 40], but the latter has also been reported previously in CLL, where both these mutations are suggested to be late-occurring CLL driver events [3, 19]. Interestingly, the combination of *ATM* deletions and *SF3B1* mutations results in a CLL-like disease in elderly mice leading to genome instability, dysregulation of CLL-associated processes and providing mechanistic evidence for disease development [41].

We observed distinct patterns of co-occurrence or mutual exclusivity among *ATM*-mutated patients when stratified by IGHV gene SHM status. In samples harboring *ATM* mutations, trisomy 12 and *TP53* mutations were found to be mutually exclusive in the U-CLL subgroup, while instead, *SF3B1* mutations were enriched in the M-CLL subgroup. Focusing specifically on Binet A cases, which amounted to almost three-quarters of the cohort (72%, 2604 patients), isolated *ATM* mutations were a very rare finding detected only in 1.3% of cases and in 0.7% of M-CLL cases. This suggests that *ATM* mutation alone, excluding del(11q), may not be a key driver of CLL progression, in contrast to what is known about the relationship between *TP53* mutation and del(17p) [42].

In order to explore the relevance of mono- and bi-allelic abnormalities of the *ATM* gene, we evaluated the clinical impact of *ATM* aberrations by separately analyzing del(11q), *ATM* mutations, and bi-allelic *ATM* abnormalities in Binet A patients. In this analysis, any type of *ATM* abnormality resulted in inferior outcome compared to wildtype cases. More interestingly, differences became more evident when we investigated U-CLL and M-CLL subgroups separately. In Binet stage A U-CLL patients, only isolated del(11q) resulted in inferior TTFT when compared to *ATM*-wildtype cases. Here, bi-allelic *ATM* abnormalities were not significant, possibly explained by the relatively low number of cases. On the contrary, the same analysis in M-CLL cases revealed instead that any *ATM* abnormality, either mono-or bi-allelic, resulted in significantly shorter TTFT. Of note, harboring multiple *ATM* mutations compared to a single mutation did not result in inferior TTFT in the entire cohort or in Binet A cases only. Additionally, TTFT analysis revealed no differences when comparing somatic missense to nonsense/frameshift mutations, when investigating C- versus N-terminal mutations or analyzing mutations in *ATM* with or without an additional *SF3B1* mutation.

As most patients with *ATM* mutations also carried other genetic alterations, we assessed the specific contribution of *ATM* mutations, independent of del(11q), on TTFT by employing a multivariable model in Binet A cases stratified by IGHV status. We found that in U-CLL, del(11q) and not *ATM* mutations were an independent marker of TTFT, suggesting that *ATM* mutations alone do not independently contribute to disease progression. Notably, del(11q) was a highly significant marker for TTFT, adding similar risk as *SF3B1* mutations or *TP53* abnormalities. For M-CLL cases, again del(11q) but not *ATM* mutations was a highly significant marker for TTFT, similar to *SF3B1* mutations. These findings highlight the prognostic impact of this aberration for both subgroups of patients. Similar findings were observed when analyzing OS, with *ATM* mutations emerging as a significant biomarker of poor prognosis in univariable models. However, in multivariable models, whether applied to the entire cohort or stratified by IGHV status, del(11q), but not *ATM* mutations, was identified as a significant predictor of OS. These latter results should however be interpreted with caution, given the substantial variability in treatment strategies among patients from the different participating centers.

The current retrospective study has several limitations which mainly arise from the multi-center composition of the cohort. Even though a significant proportion of patient samples were sequenced using targeted gene panels, a smaller subset underwent whole-genome (WGS) or whole-exome sequencing (WES) contributing to methodological variability. Additionally, for cases sequenced using NGS-based techniques, sequence alignment, variant calling, annotation and initial variant filtering were conducted independently at each participating center. Moreover, most samples were derived from unsorted peripheral blood mononuclear cells (PBMCs), and although unpurified CLL samples generally have high tumor content (>80%), mutations with VAFs near the 5% threshold could be missed, potentially underestimating the number of mutated cases. Finally, the proportion of cases requiring treatment seems slightly higher than in other published cohorts [43–45], likely explained by the long follow-up period (median 13 years) and the inclusion of samples from a clinical trial cohort.

In conclusion, our findings demonstrate that del(11q) significantly influences TTFT in CLL, impacting both the U-CLL and M-CLL subgroups. In contrast, somatic *ATM* mutations do not appear to have an independent prognostic impact in CLL. Isolated *ATM* mutations were rare, especially in M-CLL. Instead, *ATM* mutations often co-occurred with other poor-prognostic biomarkers, particularly del(11q) and *SF3B1* mutations. Although del(11q) is strongly associated with a shorter TTFT, patients with this genetic aberration treated with chemoimmunotherapy can overcome the poor prognosis linked to del(11q), achieving prolonged OS [10]. Similar results are observed in ibrutinib-treated patients, where those with del(11q) were suggested to have a significantly longer PFS compared to patients without del(11q) [46–48]. Furthermore, relapsed/refractory patients with CLL carrying del(11q) were also shown to benefit from the combination venetoclax-rituximab compared to those receiving bendamustine and rituximab therapy [49]. These results highlight the need for large-scale, uniform cohort studies to better understand the overall prognostic and predictive roles of not only del(11q) and *ATM* mutations, but also other recurrent genetic abnormalities in CLL. Based on current evidence, we propose that del(11q), but not *ATM* mutations, should be considered a key factor in identifying patients at high risk for early disease progression and in determining therapeutic strategies. This is particularly important in the M-CLL subgroup, where del(11q) along with other recurrent mutations can help identify outlier cases with a poor prognosis despite otherwise indolent features.

## Supplementary information

Supplemental material

Supplemental tables

## Data Availability

The data that support the findings of this study are available from the corresponding author upon reasonable request.
