# Supplementary material for: ATM aberrations in chronic lymphocytic leukemia: del(11q) rather than ATM mutations is an adverse-prognostic biomarker
Source: Leukemia. 2025 Apr 24;39(7):1650–60. doi: 10.1038/s41375-025-02615-5 (PMC12208880; doi:10.1038/s41375-025-02615-5)
Supplement: Supplementary file 1 — Supplemental material [file 41375_2025_2615_MOESM1_ESM.pdf]

Supplemental information

ATM aberrations in chronic lymphocytic leukemia: del(11q) rather than *ATM* mutations is an adverse-prognostic biomarker

Birna Thorvaldsdottir*, Larry Mansouri*, Lesley-Ann Sutton, Ferran Nadeu, Manja Meggendorfer, Helen Parker, Christian Brieghel, Stamatia Laidou, Riccardo Moia, Davide Rossi, Jana Kotaskova, Julio Delgado, Ana E Rodríguez-Vicente, Rocío Benito, Gian Matteo Rigolin, Silvia Bonfiglio, Lydia Scarfo, Mattias Mattsson, Zadie Davis, Panagiotis Baliakas, Inmaculada Rapado, Fatima Miras, Joaquín Martinez-Lopez, Javier de la Serna, Jesús María Hernández Rivas, María José Larráyoz, María José Calasanz, Karin E. Smedby, Blanca Espinet, Anna Puiggros, Lars Bullinger, Francesc Bosch, Bárbara Tazón-Vega, Fanny Baran-Marszak, David Oscier, Florence Nguyen-Khac, Thorsten Zenz, Maria Jose Terol, Antonio Cuneo, María Hernández-Sánchez, Sarka Pospisilova, Gianluca Gaidano, Carsten U. Niemann, Elias Campo, Jonathan C Strefford, Paolo Ghia**, Kostas Stamatopoulos**, Richard Rosenquist**, on behalf of the European Research Initiative in CLL (ERIC) and HARMONY Alliance.

*Contributed equally as first authors; **Contributed equally as senior authors.

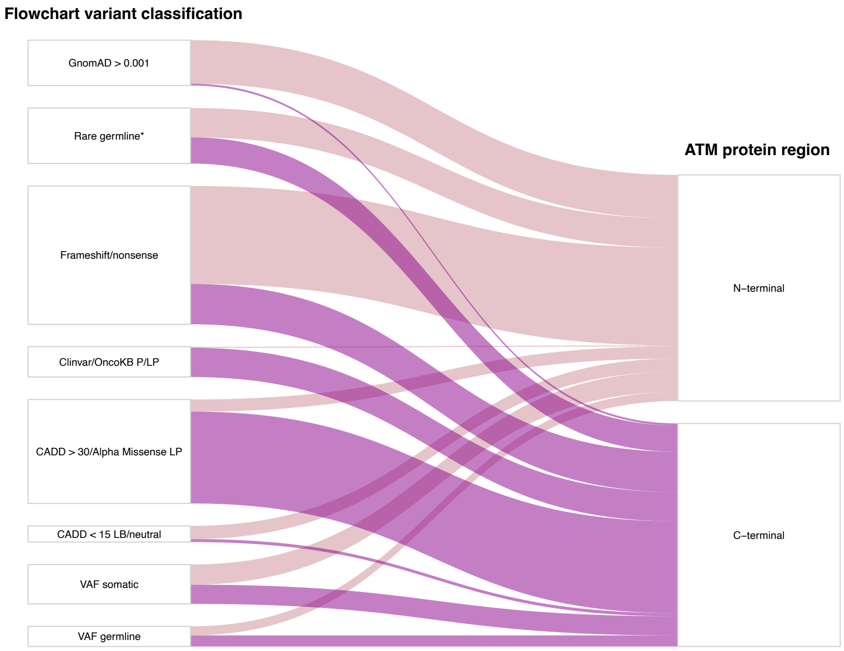

Supplemental Figure S1. Distribution of *ATM* variants in the N- versus the C-terminal of the ATM protein. Data shown for the different categories of the hierarchical classification flowchart. The C-terminal is defined as the start of the FAT-domain (UniProt).

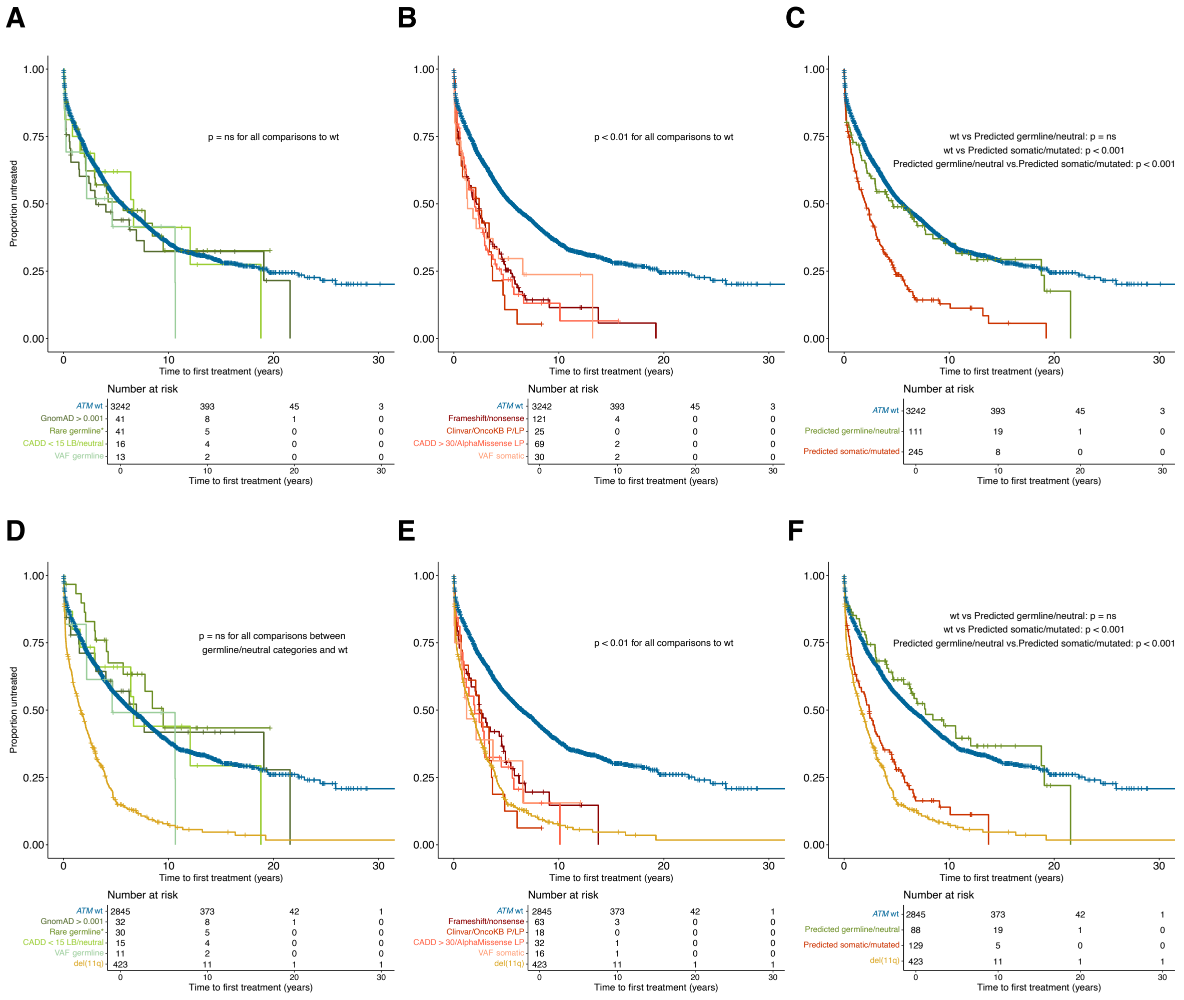

Supplemental Figure S2. Assessment of hierarchical classification of *ATM* variants in the entire cohort. A TTFT in CLL patients with *ATM* variants classified as 'germline/neutral', stratified by hierarchical flowchart categories. **B** TTFT in CLL patients with *ATM* variants classified as 'somatic/mutated', stratified by hierarchical flowchart categories. **C** TTFT in CLL patients carrying variants assigned to 'germline/neutral' categories combined and 'somatic/mutated' categories combined. **D** TTFT in CLL patients carrying *ATM* variants classified as 'germline/neutral', stratified by flowchart categories and del(11q). **E** TTFT in CLL patients carrying *ATM* variants classified as 'somatic/mutated', stratified by flowchart categories and del(11q). **F** TTFT in CLL patients carrying variants assigned to 'germline/neutral' categories combined, 'somatic/mutated' categories combined and cases with del(11q).

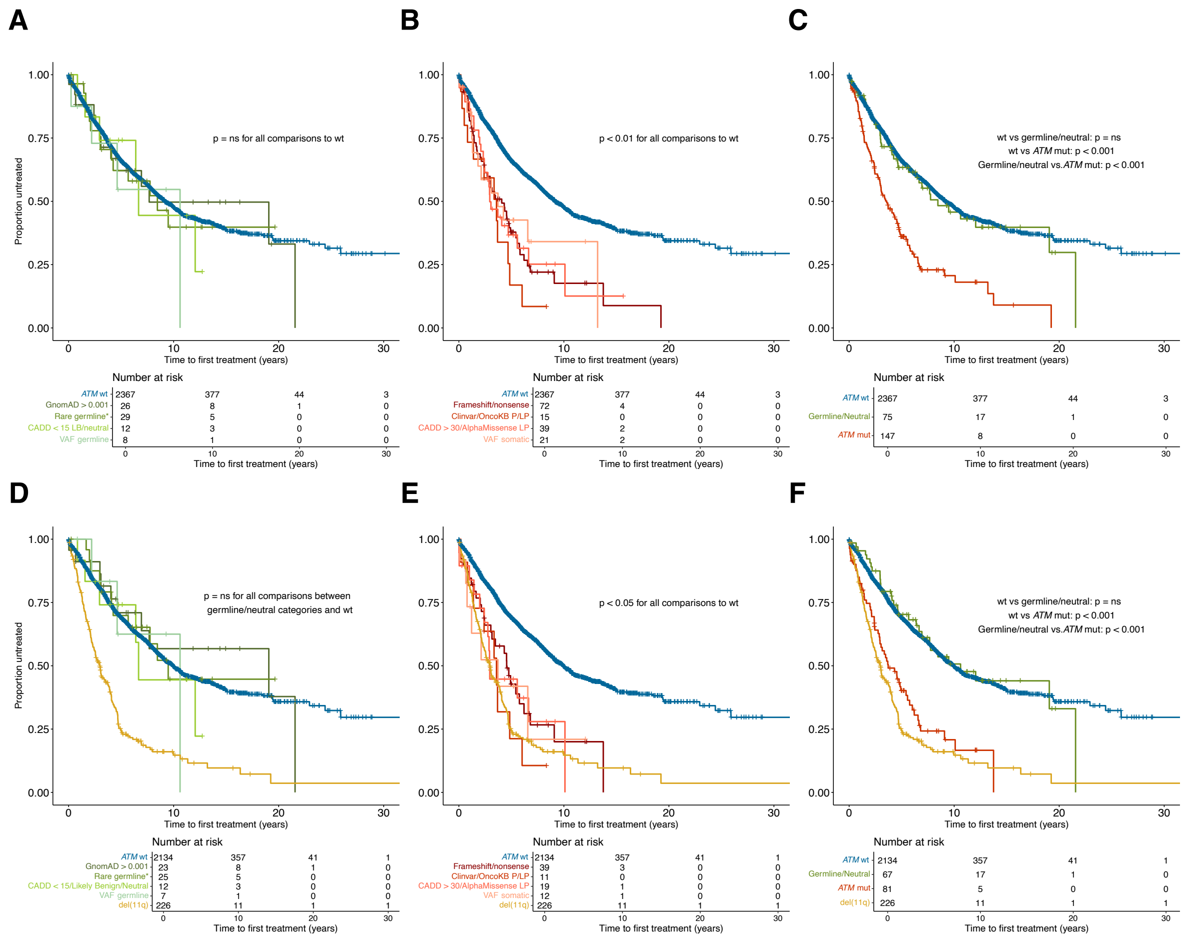

Supplemental Figure S3. Assessment of hierarchical classification of *ATM* variants in Binet A CLL patients. TTFT in Binet A CLL patients carrying *ATM* variants classified as **A** 'germline/neutral' or **B** 'somatic/mutated', stratified by flowchart categories. **C** TTFT in Binet A CLL patients carrying variants assigned to 'germline/neutral' categories combined and 'somatic/mutated' categories combined. TTFT in Binet A CLL patients carrying *ATM* variants classified as **D** 'germline/neutral' or **E** 'somatic/mutated', stratified by flowchart categories and del(11q). **F** TTFT in Binet A CLL patients carrying variants assigned to 'germline/neutral' categories combined and 'somatic/mutated' categories combined and cases with del(11q).

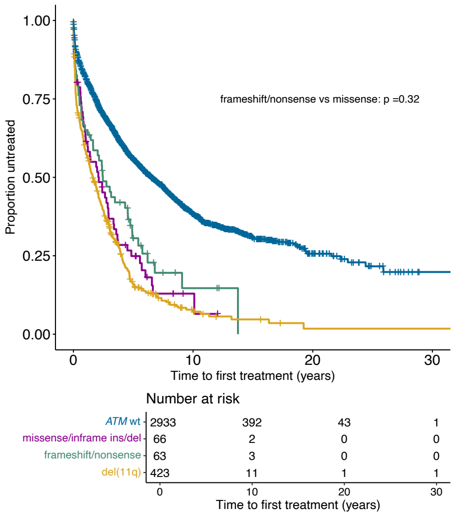

Supplemental Figure S4. Clinical effect of type of *ATM* mutation. TTFT analysis comparing type of *ATM* mutation; frameshift/nonsense, missense/inframe indels and del(11q) in all cases.

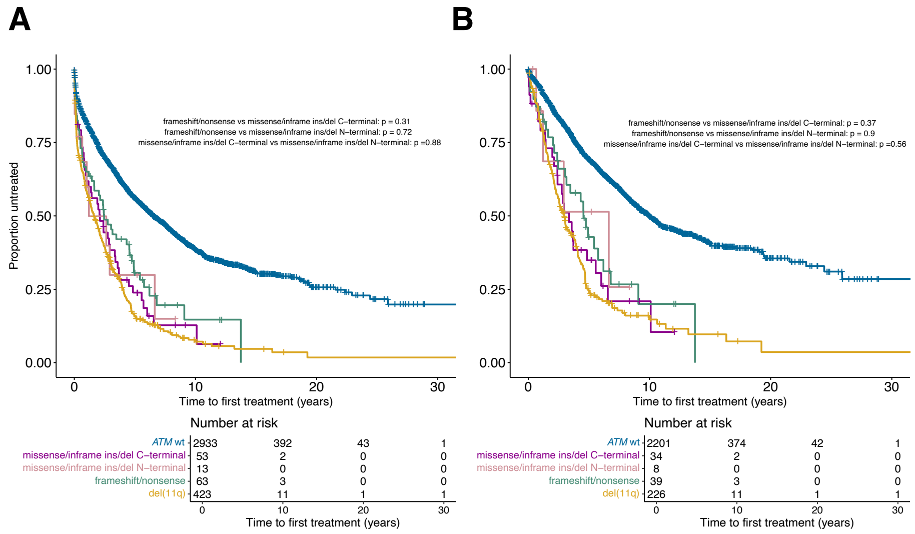

Supplemental Figure S5. Clinical effect of position of *ATM* mutation. TTFT analysis comparing position of *ATM* mutations in the C- vs N-terminal region (C-terminal region defined as the start of the FAT domain according to UniProt) and del(11q) in **A** all cases and **B** Binet A cases.

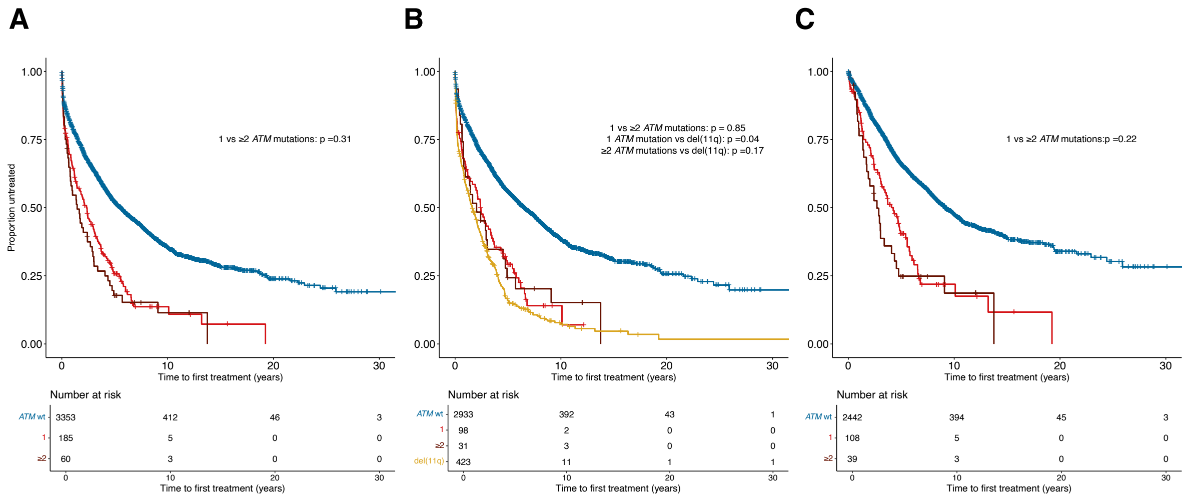

Supplemental Figure S6. Clinical effect of multiple *ATM* mutations. TTFT analysis comparing one versus multiple *ATM* mutations in **A** all patients and in **B** all patients with cases harboring del(11q) displayed separately. **C** TTFT comparing one versus multiple *ATM* mutations in Binet A patients.

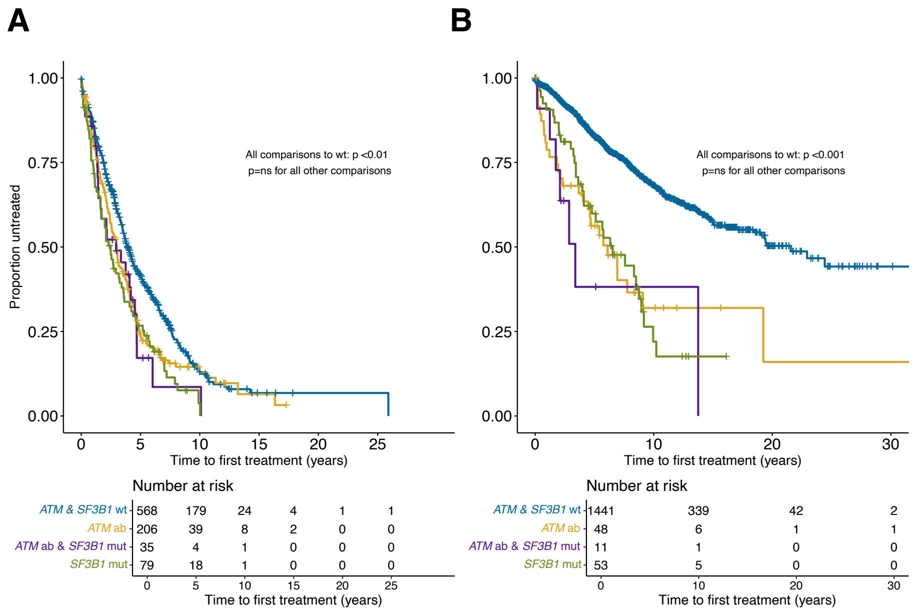

Supplemental Figure S7. Clinical effect of co-occurring *SF3B1* mutations and *ATM* aberrations. TTFT analysis in Binet A CLL patients comparing *ATM* aberrations (*ATM*ab: *ATM* mutations, del(11q) or combined *ATM* mutations and del(11q)), sole *SF3B1* mutations and combined *SF3B1* mutations and *ATM* abnormalities in **A** U-CLL and **B** M-CLL. U-CLL, CLL with unmutated IGHV genes, M-CLL, CLL with mutated IGHV genes.

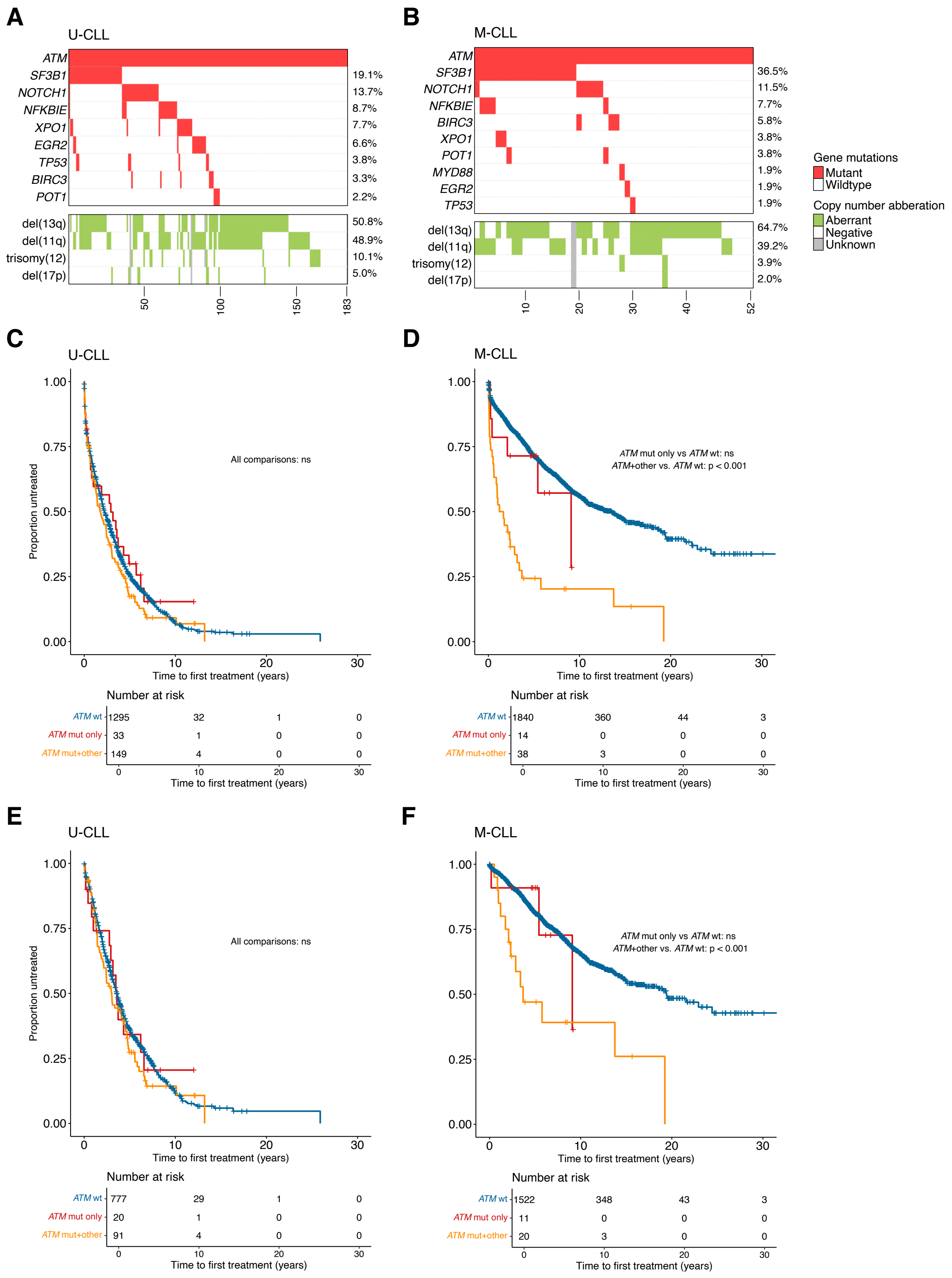

Supplemental Figure S8. Co-occurrence and clinical impact of *ATM* mutations and other genetic abnormalities. Co-occurrence of *ATM* mutations and other detected gene mutations and chromosomal aberrations in **A** 183 *ATM* mutated U-CLL cases and **B** 52 *ATM* mutated M-CLL cases. TTFT in patients carrying *ATM* mutations as sole abnormalities or in combination with other aberrations in **C** all U-CLL cases, **D** all M-CLL cases, **E** Binet stage A U-CLL cases and **F** Binet stage A M-CLL cases. Other aberrations: mutations in *BIRC3, EGR2, NFKBIE, NOTCH1,*

POT1, SF3B1, TP53, XPO1 and chromosomal aberrations: del(11q), del(17p) and trisomy 12. U-CLL, CLL with unmutated IGHV genes, M-CLL, CLL with mutated IGHV genes.

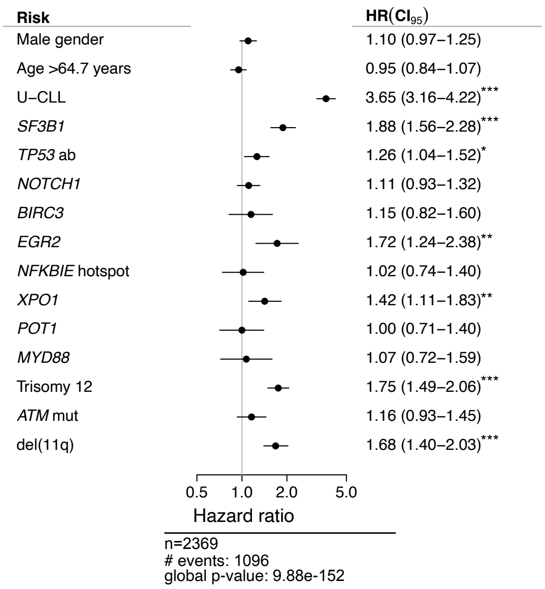

Supplemental Figure S9. Multivariable analysis of TTFT in Binet stage A CLL patients. CI95, 95% confidence interval; * indicates a p value <0.05, ** p <0.01, and *** p <0.001

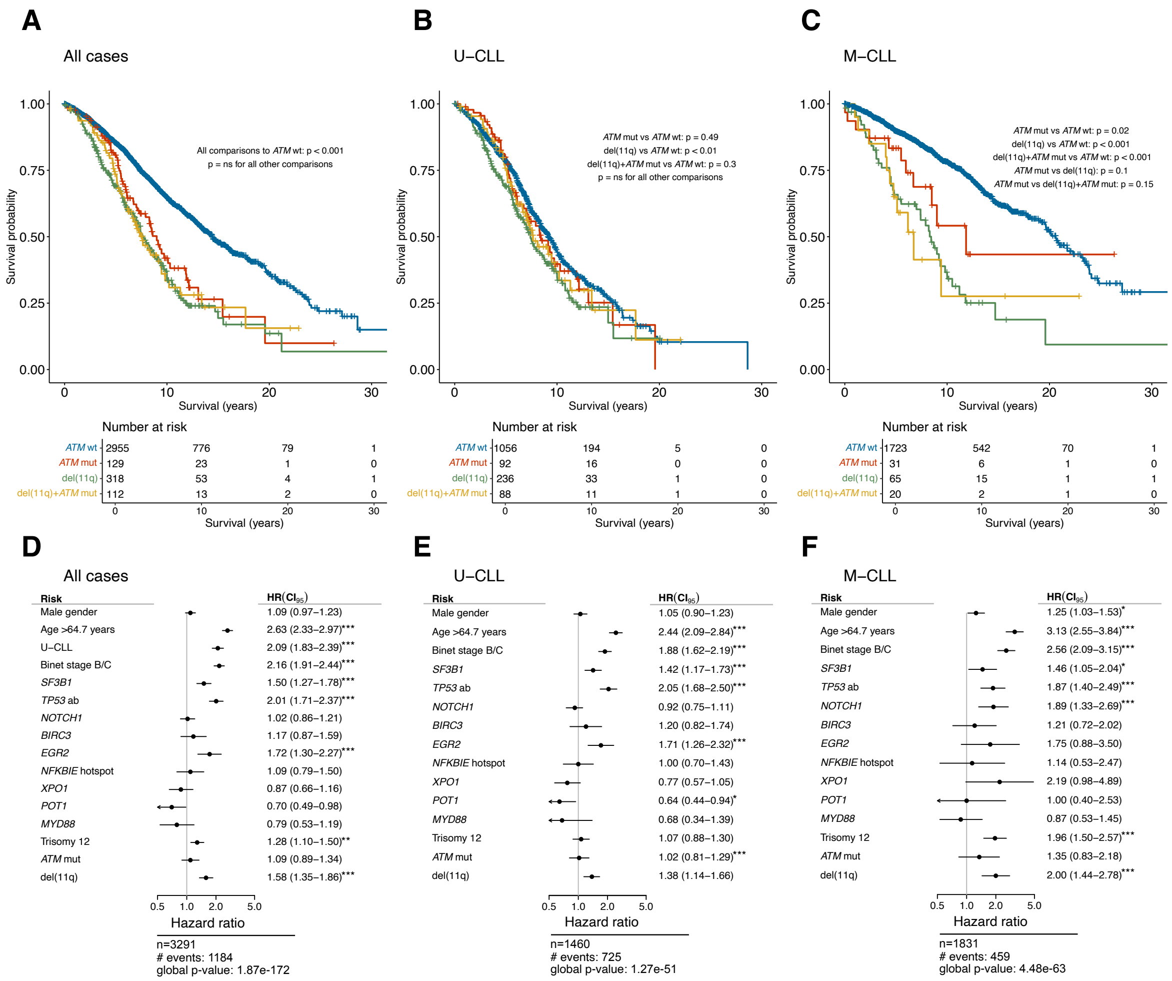

Supplemental Figure S10. Effects of *ATM* aberrations on overall survival. Kaplan-Meier survival analysis using OS subdivided by type of *ATM* aberration; somatic *ATM* mutation only, del(11q) only and combined *ATM* mutation and del(11q) in **A** all patients, **B** U-CLL and **C** M-CLL patients. Pairwise comparisons were performed using the Cox–Mantel log-rank test. Multivariable analysis in **D** all CLL patients in the cohort **E** U-CLL patients and **F** M-CLL patients. U-CLL, CLL with unmutated IGHV genes, M-CLL, CLL with mutated IGHV genes. CI95, 95% confidence interval; * indicates a p value <0.05, ** p <0.01, and *** p <0.001
